# Contribution of oxidative stress to endothelial dysfunction in hypertension

**DOI:** 10.3389/fphys.2012.00441

**Published:** 2012-12-05

**Authors:** Bruno R. Silva, Laena Pernomian, Lusiane M. Bendhack

**Affiliations:** ^1^Department of Pharmacology, School of Medicine of Ribeirão Preto, University of São Paulo, Ribeirão PretoSão Paulo, Brazil; ^2^Department of Physics and Chemistry, Faculty of Pharmaceutical Sciences of Ribeirão Preto, University of São Paulo, Ribeirão PretoSão Paulo, Brazil

**Keywords:** oxidative stress, endothelial dysfunction, hypertension, vascular relaxation, vascular contraction, NO availability

## Abstract

Endothelial dysfunction is the hallmark of hypertension, which is a multifactorial disorder. In the cardiovascular system reactive oxygen species play a pivotal role in controlling the endothelial function and vascular tone. Physiologically, the endothelium-derived relaxing factors (EDRFs) and endothelium-derived contractile factors (EDCFs) that have functions on the vascular smooth muscle cells. The relaxation induced by the EDRFs nitric oxide (NO), prostacyclin, and the endothelium-derived hyperpolarization factor (EDHF) could be impaired in hypertension. The impaired ability of endothelial cells to release NO along with enhanced EDCFs production has been described to contribute to the endothelium dysfunction, which appears to lead to several cardiovascular diseases. The present review discusses the role of oxidative stress, vascular endothelium, and vascular tone control by EDRFs, mainly NO, and EDCFs in different models of experimental hypertension.

Hypertension is a multifactorial disorder that involves many mechanisms leading to risk factors for cardiovascular diseases. Endothelial dysfunction is defined as the imbalance between the production and bioavailability of endothelium-derived relaxing factors (EDRFs) and endothelium-derived contractile factors (EDCFs), associated with increased bioavailability of oxygen reactive species (ROS) and decreased antioxidant capacity characterized as oxidative stress. In this review we will discuss the involvement of oxidative stress and vascular endothelium as well as the importance of vascular tone control, relaxation, and contraction in hypertension.

NO is an important mediator released by endothelial cells. It is produced by NO synthases (NOS), which convert L-arginine and molecular oxygen to L-citrulline and NO, using such co-factors as tetrahydrobiopterin (BH_4_), flavin-adenine-dinucleotide, flavin-mononucleotide, and nicotinamide-adenine-dinucleotide-phosphate (Thomas et al., 2008). The activity of NOS is regulated by substrate, cofactor availability, and electron transfer rate. The regulating factors such as arginine (Gornik and Creager, 2004) and BH_4_ (Bevers et al., 2006) can be affected by ROS that can lead to dysfunctional eNOS. As summarized in the Figure 1, in pathological states involving oxidative stress such as hypertension NOS could be uncoupled (Schulz et al., 2008). L-arginine is the substrate for both enzymes, NOS and arginase (Tousoulis et al., 2002). Zhang et al. (2004) showed that the activity of arginase in the endothelial cells of coronary arterioles is increased in hypertension, which impairs the NO-mediated dilation. Similarly, as reported by Chandra et al. (2012) peroxynitrite (ONOO^−^) and hydrogen peroxide (H_2_O_2_) increase arginase activity/expression in the endothelial cells. This should lead to NOS uncoupling with reduced NO production and augmented superoxide anion (O^−^_2_) production. As shown by Romero et al. (2008), increased arginase activity in diabetes contributes to vascular endothelium dysfunction by decreasing L-arginine availability to NOS.

**Figure 1:**
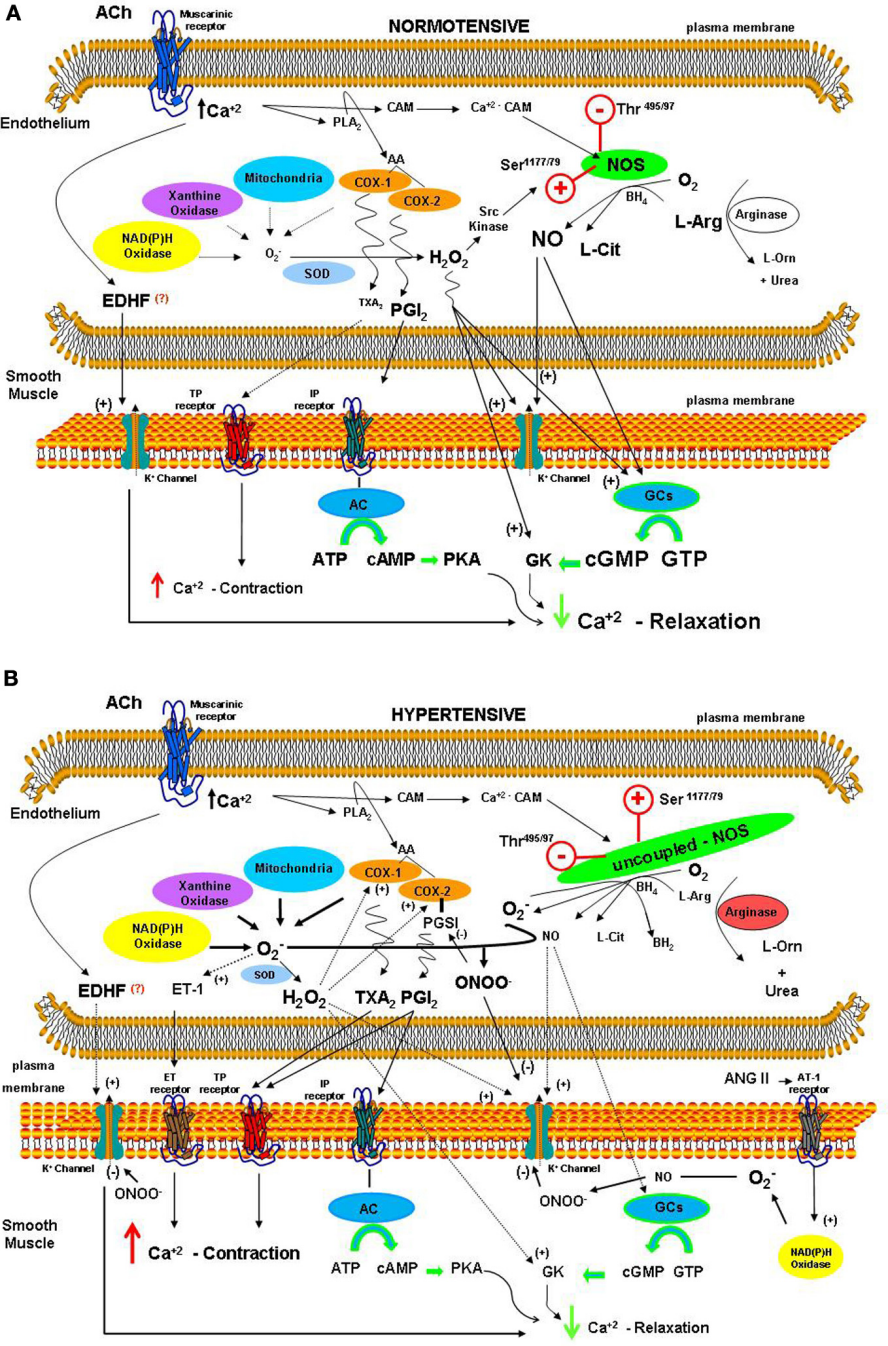
**Sources of reactive oxygen species (ROS) and proposed mechanisms for their contribution to EDRFs and EDCFs releasing involved in the control of vascular tone in isolated vessels from normotensive (A) and hypertensive (B) animals**.

Endothelial dysfunction culminates in impaired endothelium-dependent relaxation due to decreased vascular NO bioavailability caused by ROS consumption. The result is ONOO^−^ formation, lower NOS protein expression, or lack of substrate or co-factor for NOS (Crimi et al., 2007). The eNOS phosphorylation state can alter its activity; i.e., Akt-dependent phosphorylation at Ser^1177^ (human) or Ser^1179^ (bovine) activates eNOS (Fulton et al., 1999), while phosphorylation at Thr^495^ (human) or Thr^497^ (bovine) decreases its activation (Bouloumié et al., 1997). H_2_O_2_ initially raises eNOS Ser^1179^ phosphorylation and activity, in parallel with transient Akt activation (Hu et al., 2008).

*In vivo* measurements of NO and H_2_O_2_ in the mesenteric arteries of spontaneously hypertensive rats (SHR) revealed higher baseline NO and H_2_O_2_ concentrations than normotensive rats (Zhou et al., 2008). It is known that in resistance arteries more than in conduit vessels, EDHF is an important control of vascular tone. H_2_O_2_ has been shown to be a component of EDHF in several vascular beds (Meurer et al., 2005; Shimokawa, 2010; Prysyazhna et al., 2012).

Peroxynitrite can also activate eNOS by increasing basal and agonist-stimulated Ser^1179^ phosphorylation, although it reduces NO bioavailability and elevates O^−^_2_ production (Zou et al., 2002a). eNOS exposure to oxidants like ONOO^−^ causes increased enzymatic uncoupling and O^−^_2_ generation in diabetes that contributes to endothelial cell oxidant stress (Zou et al., 2002b). Increased formation of ONOO^−^ can inhibit prostacyclin synthase (PGIS) (Wu and Liou, 2005) and impairs K^+^ channel activation (Gutterman et al., 2005).

Increased ROS bioavailability, decreased antioxidant capacity, or both occur in many models of hypertension such as SHR (Suzuki et al., 1995), Dahl salt-sensitive (Swei et al., 1997), AngII-infused rats (Laursen et al., 1997), renal hypertensive 2K-1C (Rodrigues et al., 2008), and human hypertension (Vaziri, 2004). In endothelial cells, the ROS producers are NADPH oxidase (Rajagopalan et al., 1996), xanthine oxidase (Phan et al., 1989), uncoupled NOS (Satoh et al., 2005), cyclooxygenase (COX) (Tang et al., 2007), and mitochondria (Callera et al., 2006). The DOCA-salt model present augmented oxidative stress caused by increased NADPH oxidase activity, which accounts for enhanced O^−^_2_ production (Beswick et al., 2001). In 2K-1C rats, the increased vascular O^−^_2_ is secondary to a protein kinase C (PKC)-mediated activation of NADPH oxidase (Heitzer et al., 1999). However, eNOS activity is reduced by phosphorylation of the Thr^495^ residue in the Ca^2+^/CaM binding domain by PKC (Mount et al., 2007). Mimicking of Thr^495^ dephosphorylation results in eNOS uncoupling and O^−^_2_ production rather than NO generation (Lin et al., 2003). However, whether the Thr^495^ eNOS phosphorylation site is more phosphorylated in hypertension or contains uncoupled eNOS remains unknown.

We have investigated the vascular mechanisms involved in the vasorelaxation induced by NO donors that present potential capacity to replenish vascular NO upon reduced NO bioavailability. Most of the studies using NO donors are performed on endothelium-denuded arteries to avoid interference of endogenously produced NO (Bonaventura et al., 2004; Pereira et al., 2011). Impaired 2K-1C rat aorta relaxation is endothelium-dependent (Callera et al., 2004) or endothelium-independent (Bonaventura et al., 2005). Vitamin-C normalized the impaired relaxation induced by a NO donor in 2K-1C rat aorta that shows the increased ROS production in the vascular smooth muscle cells (Rodrigues et al., 2008). Interestingly, the endothelium can contribute to the vasorelaxation induced by sodium nitroprusside (SNP) via NOS activation (Bonaventura et al., 2008). The endothelium negatively modulates the vasorelaxation induced by the complex (TERPY) in the rat aorta. BH_4_ supplementation reverses the effect of uncoupled NOS induced by TERPY (Bonaventura et al., 2009).

The altered function of endothelial cells leads to enhanced contraction (Endemann and Schiffrin, 2004). The EDCFs released under different stimuli include ET-1 (Taddei et al., 2003), some prostanoids, and ROS (Tang and Vanhoutte, 2009). ET-1 activates ET_A_ and ET_B_ receptors. ET_A_ receptors are expressed on smooth muscle cells and promote contraction. ET_B_ receptors are located on endothelial and smooth muscle cells, with opposite effects. Smooth muscle ET_B_ activation evokes contraction, whereas endothelial ET_B_ activation induces relaxation (Taddei et al., 2003). The imbalance in the expression of receptors or increased ET-1 production can contribute to hypertension. Hypertension associated with elevated levels of AngII leads to high vascular ET-1 production (Dohi et al., 1992) as well as ROS originated from NADPH oxidase (Touyz et al., 2002). Both factors are related to larger contractility in hypertensive rat resistance arteries.

The SHR aorta exhibits a characteristic endothelial dysfunction that is not due to decreased EDRF release, but it is the result of simultaneous EDCF release. Indomethacin, a non-selective COX inhibitor, restores the blunted relaxation in SHR aorta to the level of normotensive (Lüscher and Vanhoutte, 1986), which suggests that this EDCF must be a product of the COX. Endothelium-dependent contraction is reported in the rat aorta, mesenteric and femoral arteries, and cerebral arterioles. It occurs in healthy animals, but EDCF release is exacerbated by hypertension. Selective COX-1 inhibitors abolish endothelium-dependent contraction in SHR aorta, while selective COX-2 inhibitors only display modest responses (Tang and Vanhoutte, 2009).

Endoperoxides, PGI_2_, TXA_2_, and ROS are proposed as COX-derived EDCFs. Increased endothelial [Ca^2+^]_i_ is required to evoke EDCF-mediated responses. Dysfunction in Ca^2+^ handling within the endothelium is important for the exacerbation of endothelium-dependent contractions in SHR aorta (Tang et al., 2007).

Independent of the genesis of hypertension, specific ROS such as H_2_O_2_ modify the vascular activity of NOS and COX in concentration-dependent way (Cai et al., 2003; Gil-Longo and González-Vásquez, 2005). In hypertension, ROS are involved in augmented EDCFs and diminished EDRFs release. In the L-NAME (Qu et al., 2010) and SHR (Félétou et al., 2009) models there is increased COX-derived production of contractile prostanoids. Physiologically, PGI_2_ evokes vasorelaxation, whereas in aging animals or SHR it induces contraction (Vanhoutte, 2011).

Inhibitors of COX (Taddei et al., 1997), NADPH oxidase (Costa et al., 2009), and xanthine oxidase (Ellis et al., 1998) or antioxidant agents such as Vitamin-C (Nishi et al., 2010) seem to diminish ROS production and EDCFs generation.

In conclusion, the data presented in this work suggest that decreased NO availability along with enhanced EDCFs production contribute to the endothelium dysfunction and impaired vascular relaxation in hypertension (Figure 1). Considering the enormous progress in the area in the last years, this work addresses the function of oxidative stress on the pathogenesis of hypertension.

## Conflict of interest statement

The authors declare that the research was conducted in the absence of any commercial or financial relationships that could be construed as a potential conflict of interest.
